# Pure hydrogen low-temperature plasma exposure of HOPG and graphene: Graphane formation?

**DOI:** 10.3762/bjnano.3.96

**Published:** 2012-12-13

**Authors:** Baran Eren, Dorothée Hug, Laurent Marot, Rémy Pawlak, Marcin Kisiel, Roland Steiner, Dominik M Zumbühl, Ernst Meyer

**Affiliations:** 1 Department of Physics, University of Basel, Klingelbergstrasse 82, CH-4056, Basel, Switzerland

**Keywords:** graphane, HOPG, hydrogenation, plasma

## Abstract

Single- and multilayer graphene and highly ordered pyrolytic graphite (HOPG) were exposed to a pure hydrogen low-temperature plasma (LTP). Characterizations include various experimental techniques such as photoelectron spectroscopy, Raman spectroscopy and scanning probe microscopy. Our photoemission measurement shows that hydrogen LTP exposed HOPG has a diamond-like valence-band structure, which suggests double-sided hydrogenation. With the scanning tunneling microscopy technique, various atomic-scale charge-density patterns were observed, which may be associated with different C–H conformers. Hydrogen-LTP-exposed graphene on SiO_2_ has a Raman spectrum in which the D peak to G peak ratio is over 4, associated with hydrogenation on both sides. A very low defect density was observed in the scanning probe microscopy measurements, which enables a reverse transformation to graphene. Hydrogen-LTP-exposed HOPG possesses a high thermal stability, and therefore, this transformation requires annealing at over 1000 °C.

## Introduction

**Figure 1:**
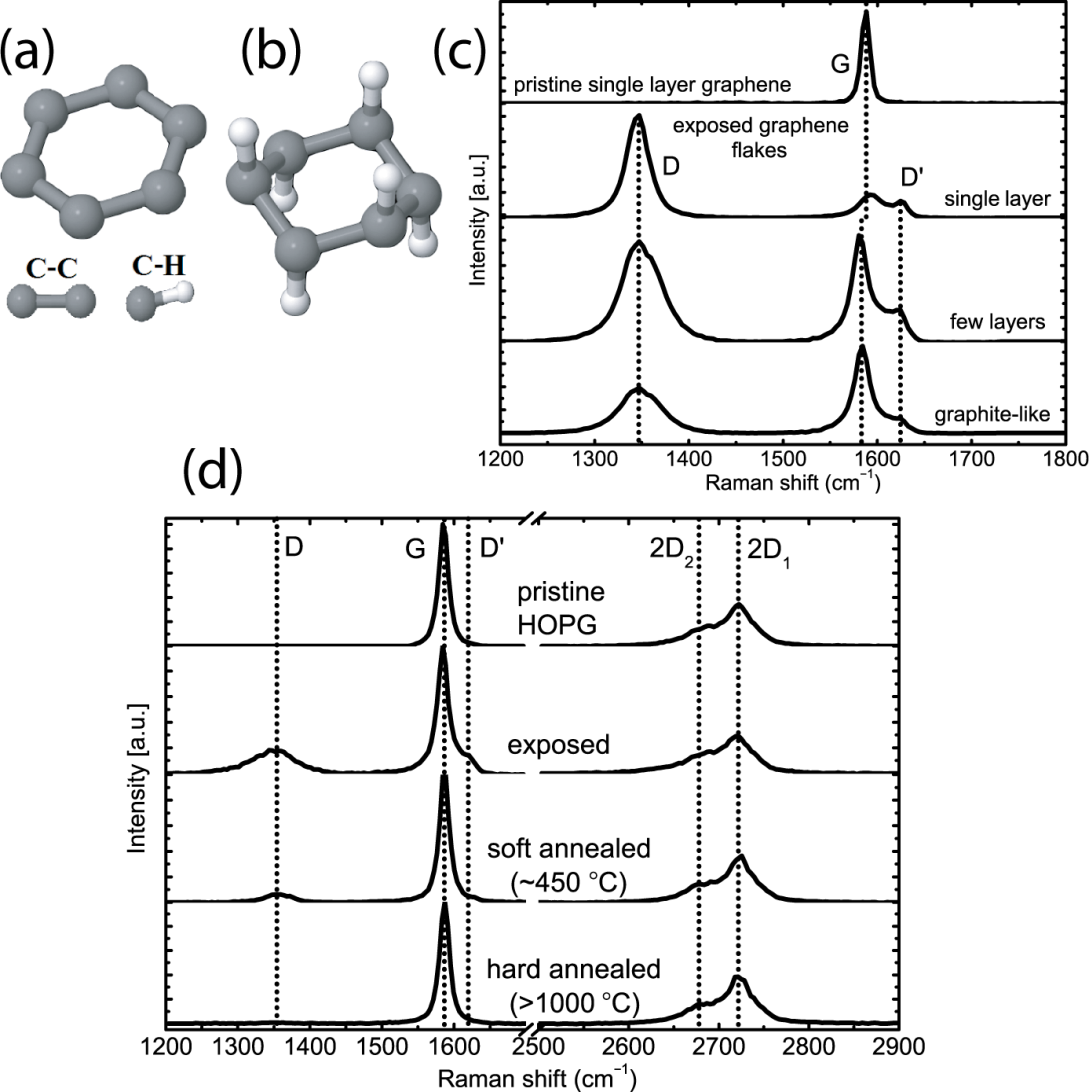
The two-dimensional material consisting of carbon atoms in honeycomb orientation, graphene (a), loses its sp^2^ hybridization upon hydrogenation from both sides resulting into its insulating derivative, graphane (b). This transformation may be achieved with a pure hydrogen LTP exposure, which results in a D/G height ratio of 4.5 in the Raman spectrum of single layer graphene (c). The sample was kept at 450 °C during 5 min of exposure, where the same plasma exposure results in a smaller D/G ratio for multilayer graphene (c) and for HOPG (d), due to contributions from pristine layers beneath the hydrogen implantation depth. Soft annealing at 450 °C diminishes the D and D' peaks; however a complete suppression, which signifies reversibility to graphene, is achieved only after an annealing over 1000 °C.

Being an sp^2^-hybridized single layer of carbon atoms arranged in a densely packed honeycomb lattice with true atomic thickness (Figure 1a), graphene possesses unusual electronic and mechanical properties [1–2]. A new perspective is the chemical modification of graphene, especially the incisive idea of attaching atomic hydrogen to both sides of the graphene lattice to produce graphane (Figure 1b): an sp^3^-hybridized insulating derivative of graphene [3–6]. Graphane offers a brand new playground for physicists and engineers, particularly as a prospect for two-dimensional electronic applications. Nanowire [7] or transistor concepts consisting of only graphene and graphane could be realized. Another possible application is based on its characteristics in terms of hydrogen storage. It has a volumetric capacity of 0.12 kg H_2_/L, which is higher than the Department of Energy target of 0.081 kg H_2_/L for the year 2015 [3].

A prerequisite for graphane synthesis is the abundance of atomic hydrogen to react with unsaturated C–C bonds of graphene; subsequently leading to C–H bond formation on both sides of the graphene. The elegant yet simple solution to obtain such a chemisorption may be to use a pure hydrogen low-temperature plasma (LTP) with a typical average electron temperature (*T*_e_) of 2–5 eV, where the hydrogen would be easily dissociated (with the required energy being *T*_e_: 4.52 eV) and chemisorbed on the surface, and a small portion will be ionized (required energy *T*_e_: 13.6 eV). The atomic ion implantation may also hydrogenate the other side of the surface layer and even some other subsurface layers. The 3.5 eV plasma used in this work results in an ion impact energy (ε_i_) of 12.6 eV on the sample surface. With this technique, proton deposition energies can be obtained that are high enough to overcome the energy barrier (3.7 eV) to penetrate the center of the hexagonal carbon [8], without physically sputtering (36 eV) the carbon atoms [9–10]. Moreover, hydrogen ions can even be implanted deeper into the first 4–5 layers of HOPG, suggested by the SRIM simulations [11].

The question is: Will graphane form after the plasma exposure and if it does, is it possible to distinguish its existence from other possible surface rearrangements caused by the exposure? Before starting, it should be clearly stated that graphane is a reserved word for graphene that is fully hydrogenated from both sides. In reality, there will always be hydrogen deficiencies and point defects and the obtained material would not be a perfect graphane. Therefore, the term hydrogenated graphene will be used for the rest of this article referring to a graphane-like structure.

The interaction of hydrogen with graphitic surfaces had been investigated a number of times in the past few decades. The earlier research concentrated on the physisorption of hydrogen molecules on such surfaces [12–14]. This was followed by theoretical [15], and experimental works focused on the chemisorption of atomic hydrogen [16–20]. A new research focus is the investigation of hydrogen-containing plasmas with graphitic surfaces [5,21]. Particularly the work of Elias et al. is interesting, in which graphane growth was claimed after exposure of free-standing graphene to a plasma containing 10% hydrogen [5]. In this work, single and multilayer graphene and highly ordered pyrolytic graphite (HOPG) were exposed to a pure hydrogen LTP, and various techniques such as photoelectron spectroscopy, Raman spectroscopy and scanning probe microscopy were employed for characterization. However, due to the insufficient electrical conductance, it was not possible to use photoelectron spectroscopy and scanning tunneling microscopy techniques for graphene on SiO_2_.

In contrast to plasma treatments in previous works [5,21], mixing of a second gas was avoided in this work. Introduction of a high-*Z* gas, such as argon, would have cooled down the plasma due to the increasing number of recombinations, which would have hindered hydrogen implantation. This may be the reason why graphane formation was claimed only for free-standing graphene by Elias et al. [5]. Moreover, low-energy argon ions also result in changes in the atomic structure of HOPG and, therefore, are not desired in this work [22].

## Results and Discussion

### Raman spectroscopy

Raman spectroscopy is a frequently used tool for the analysis of graphitic materials. The Raman spectrum of graphite consists of D and G peaks, around 1350 cm^−1^ and 1585 cm^−1^ respectively, which arise from vibrations of sp^2^-hybridized carbon atoms [23–26]. The D peak is caused by breathing-like modes corresponding to transverse optical phonons near the K point of the Brillouin zone. It is an intervalley double-resonance Raman process that is initiated only by a deviation from the defectless two-dimensional character [23–24,26]. On account of this, both hydrogenation and any kind of disorder manifest themselves as the rise of this peak, and a distinction between these two phenomena is not possible. Its overtone, the 2D peak that appears around 2700 cm^−1^, is a second-order process involving two inelastic scatterings, and it is always present. The shape of this 2D peak is defined by the number of graphene layers (i.e., two peaks at 2682 cm^−1^ and 2723 cm^−1^ for graphite, but a single peak at 2671 cm^−1^ for single-layer graphene) [25]. The G peak represents the optical E_2_*_g_* phonons at the center of the Brillouin zone. The cross-section for the C–C sp^3^ vibrations, when available, is negligible for visible excitation.

Upon hydrogen plasma exposure of single-layer graphene (Figure 1c, 2nd panel), a sharp D' peak around 1620 cm^−1^ appears as a result of an intervalley double-resonance process due to deviation from the defectless two-dimensional character, as well as a D peak around 1350 cm^−1^. The G peak preserves its position at 1585 cm^−1^ and a significant broadening is not observed. The sharpness of these peaks signifies that amorphization is negligible [24]. A D/G height ratio of 4.5 is observed, which suggests a strong atomic rearrangement. In the work of Elias et al. [5], such a D/G ratio, being almost twice as much as of the ratio obtained after single-surface hydrogenation, was interpreted as graphane formation. In contrast to this, the plasma exposure on HOPG results in the appearance of the D and D' peaks with lower relative intensities (Figure 1d, 2nd panel), which we attribute to the contribution of the bulk layers where no hydrogenation takes place. This assumption is consolidated with the observation of a decreasing D/G ratio for an increasing number of graphene layers that were simultaneously exposed to a hydrogen plasma (Figure 1c, 3rd and bottom panels). The plasma exposure time between 5 and 60 min did not result in a significant difference in the Raman spectrum of the HOPG (not shown here). The D and D' peaks of the Raman spectrum of the hydrogen plasma exposed HOPG can be diminished or completely suppressed by annealing at 450 °C (soft annealing) for 30 min or over 1000 °C for 10 min (hard annealing) (Figure 1d, 3rd and bottom panels, respectively). All annealings were performed under ultrahigh vacuum (UHV) conditions. The latter value is around 200 °C lower than the theoretical calculations [27], which predicts a full transformation from graphane back to graphene. In contrast to [5], we did not observe a significant change of the Raman spectrum of LTP-exposed single-layer graphene after it had been annealed at 400 °C for 24 h in an argon atmosphere.

### Atomic force microscopy

**Figure 2:**
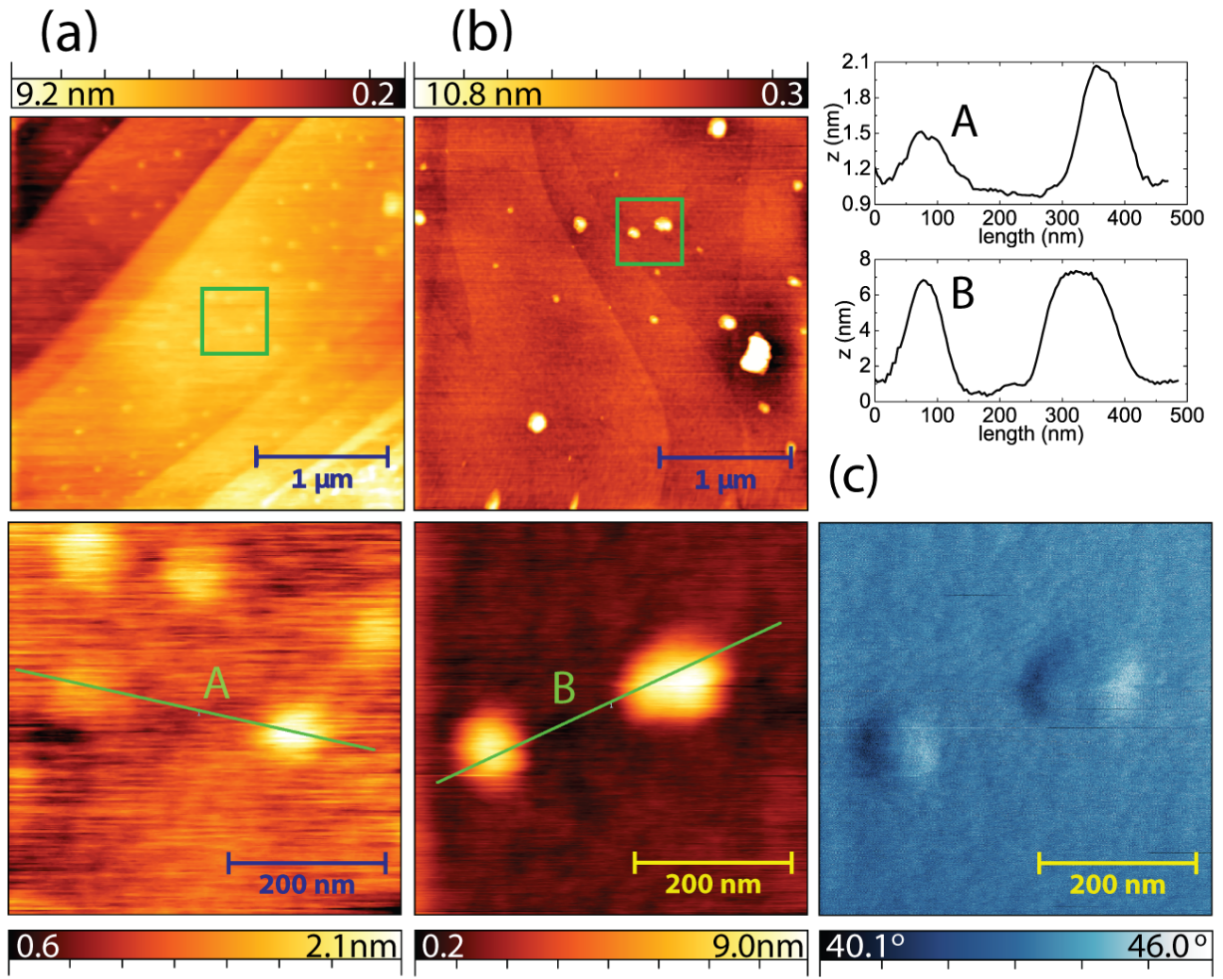
AFM measurements of HOPG after hydrogen plasma exposure at 450 °C, where round-shaped blisters appear on the topography images of the HOPG surface, with a relative height depending on the plasma exposure time ((a) 5 min, (b) 60 min). Line profiles of two blisters in (a) and (b) are presented as A and B. Blisters also reveal a phase-contrast different from the rest of the surface (c).

Though Raman spectroscopy is a strong tool for the analysis of graphitic materials, it does not provide direct evidence of hydrogenation. In order to understand the nature of the D and D' peaks of the Raman spectra, atomic force microscopy (AFM) and photoemission spectroscopy of HOPG were conducted before and after exposing it to plasma. Although the pristine HOPG exhibits a relatively flat surface, the hydrogen-plasma-exposed HOPG shows two important differences: (i) The surface becomes rougher, and (ii) blisters start to form, which are more pronounced for longer plasma exposures (Figure 2a and Figure 2b). Regarding these AFM measurements, it is clear that both surface roughening and blister formation contribute to D and D' peaks of the Raman spectrum. The surface roughening can neither be attributed to the physical sputtering, since the impact energy of the ions is well below the theoretical threshold (ε_i_ ≈ 36 eV) [9–10], nor to the chemical etching, which would have formed large hexagonal pits [28–29]. We think that some of the implanted hydrogen (including molecular ions) recombines to form hydrogen gas intercalated between two graphite layers, which builds up mechanical stress and deforms the entire surface. Moreover, this gas is free to diffuse in the lateral direction between the layers [30], which results in accumulation of hydrogen gas at certain points. Subsequently, the graphite layers start to deform more rigorously and blisters start to appear on the surface. The different phase contrast of the blisters from the rest of the surface suggests that they have different local elastic properties than elsewhere on the HOPG (Figure 2c). It is, however, not clear whether these blisters still contain hydrogen gas underneath them during storage of HOPG under ambient conditions. Similar blister formation was observed after thermal sorption of hydrogen into graphite, and hydrogen gas storage was claimed by thermal desorption experiments [31]. AFM topography images of single layer graphene on SiO_2_ do not reveal any significant roughening or blister formation (not shown here). The changes in the Raman spectrum of graphene are solely due to atomic rearrangements, either as a result of hydrogenation or corrugation at the atomic level. Since it was shown that low-energy argon-plasma treatment also results in similar atomic rearrangements due to corrugation [22], but no rise of the D peak [5], it can be claimed that hydrogen plasma treatment of the graphene layer results in its hydrogenation.

### Photoelectron spectroscopy

**Figure 3:**
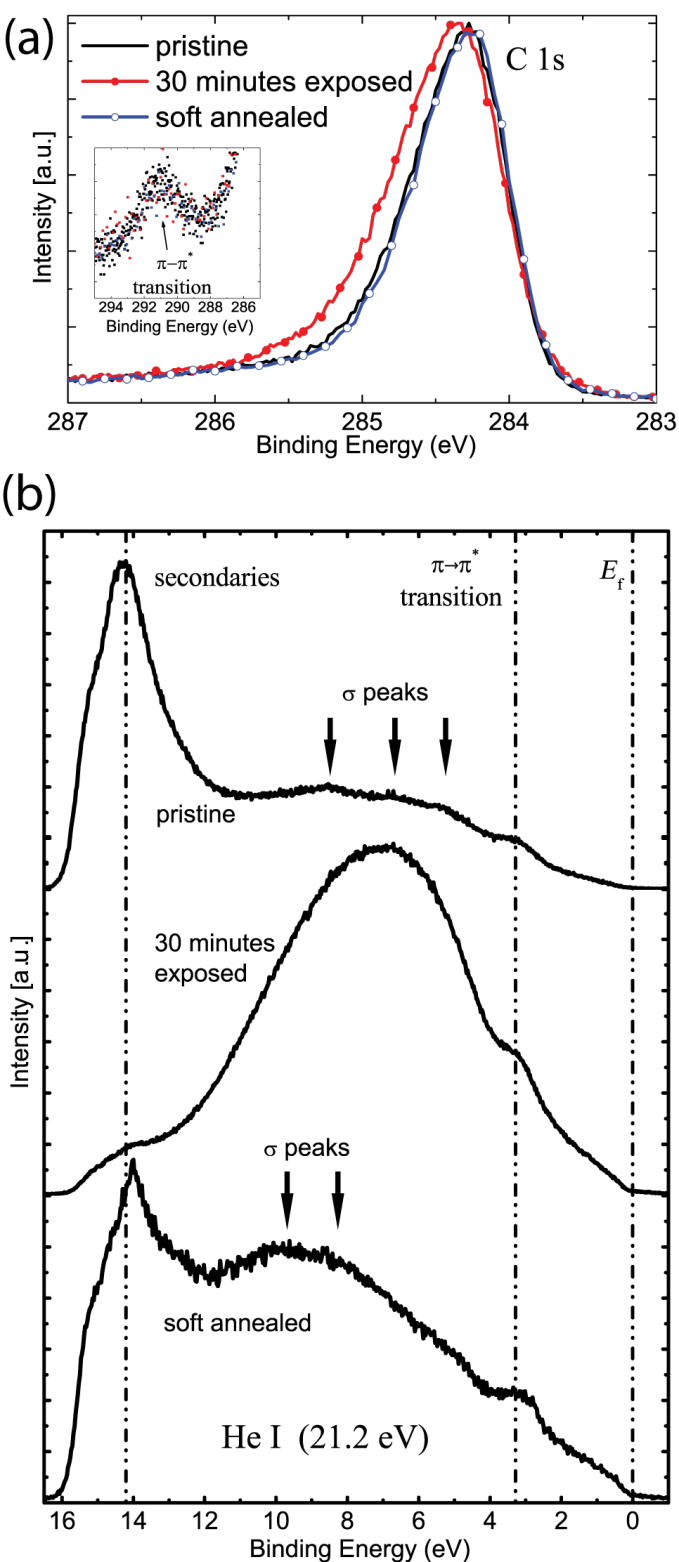
(a) XPS and (b) UPS spectra of the HOPG before exposure, after 30 min of exposure and after an annealing at 450 °C were used to monitor the transformation from graphene to hydrogenated graphene. In (b), the positions of the σ peaks and the eye-guides for the *E*_f_, the secondary electrons, and the π→π* transition are provided.

X-ray and ultraviolet photoelectron spectroscopy (XPS and UPS) were used to monitor the changes in the core level and valence band structures of the HOPG after plasma exposure and subsequently after soft annealing. After exposure to hydrogen plasma, the core level C 1s spectrum gets broadened due to roughening, whereas it recovers its initial width after soft annealing (Figure 3a). It was already stated that even though there is C–H bonding, this change in the shape of the C 1s peak is not due to a chemical shift as a result of this bonding, but is rather due to the increase in the difference between upper valence band edge and the Fermi level (*E*_f_) [32]. Such a broadening was also observed for HOPG that had been exposed to a low-energy argon plasma, and the reason for this was stated as geometric defects (roughening) without any contribution from vacancy formation or hydrogenation [22]. In a similar manner, the broadening of the C 1s peak in our case is also due to displacement of the carbon atoms and its recovery is due to flattening after soft annealing. The satellite peak due to π→π* transition exists for all the spectra (Figure 3a, inset).

As previously studied [32–34], the valence band spectrum of pristine HOPG (Figure 3b, upper panel) contains five peaks around 3.2, 5.3, 6.7, 8.5 and 14.2 eV below *E*_f_. The peak at a binding energy (B.E.) around 14.2 eV is a secondary peak arising from photoelectrons scattered into unoccupied states. The peak at a B.E. of 3.2 eV is attributed to the π→π* transition, which makes graphite a semimetal. The other three peaks lie where the π and σ bands overlap, but they have their contribution mainly from the σ band. After exposure to hydrogen plasma, the UPS spectrum looks very similar to the spectrum obtained 20 years ago by Ugolini et al., where HOPG was exposed to hydrogen ions of a Penning ion source [32]. Alone from this spectrum (Figure 3b, middle panel), an energetically favorable hydrogenated network of carbon atoms was suggested even at that time. The π→π* peak at 3.2 eV still exists denoting that the structure is layered. The remnant of the secondary peak indicates that the structure still consists of carbon rings. Since the UPS spectrum of graphite is highly dependent on the polar angle (i.e., the angle between the entrance slit of the analyzer and surface normal) [35], intensity contributions from different polar angles are smeared out and appear as one broad peak 7 eV below *E*_f_ as a result of surface roughening after plasma exposure. After soft annealing (Figure 3b, bottom panel), the secondary peak rises due to the flattening of the layers. The broadening effect is alleviated and the σ peaks reappear at shifted positions at 8.3 and 10.2 eV below *E*_f_. As suggested by the theoretical calculation of hydrogenated graphite from Allouche et al. [34], full hydrogenation of graphite results in a σ band structure very similar to diamond, an sp^3^ hybridized carbon allotrope [33,36]. From this point of view, we can claim that this UPS spectrum is the valence band spectrum of hydrogenated HOPG, analogous to cubic diamond. In the next section, it is shown that the surface still has slight corrugation, which could also have a small contribution to the UPS spectrum as the remnant of the broad peak at 7 eV. After soft annealing, the D and D' peaks in the Raman spectrum of the HOPG are diminished (Figure 1d, 3rd panel), where this partial suppression is due to flattening; however the contribution from the atomic rearrangement of the C atoms, which is possibly due to the C–H bonding, is still present. The shift of the σ peak positions towards sp^3^ hybridization supports this assumption.

### Scanning tunneling microscopy

**Figure 4:**
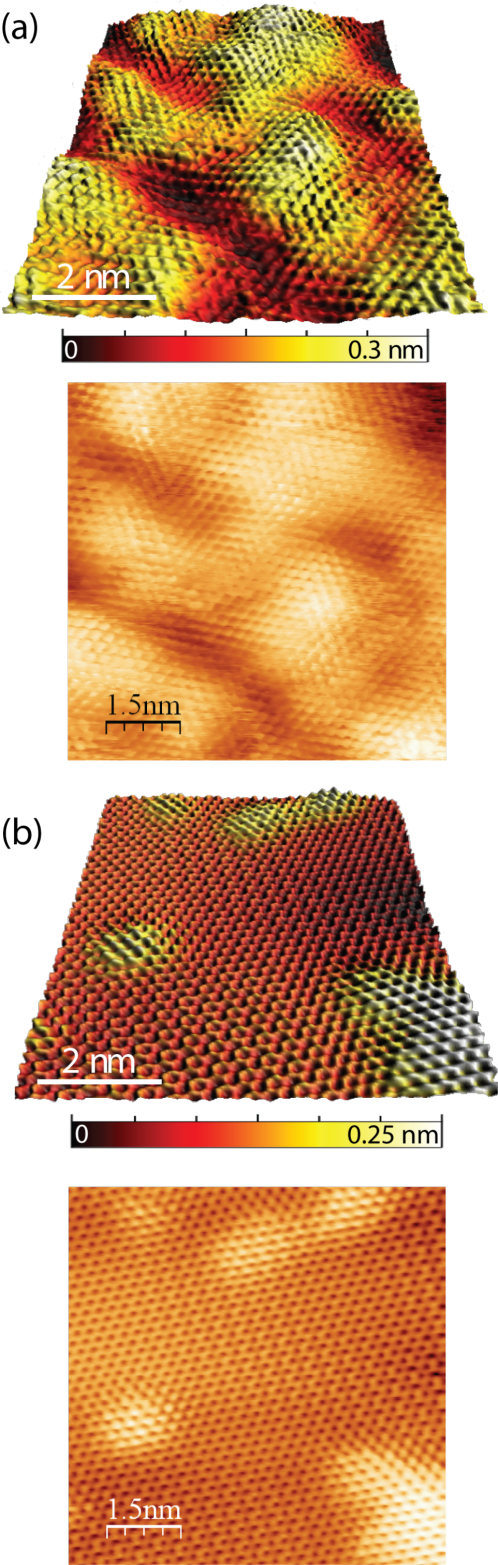
(a) STM images of the HOPG after 5 min of hydrogen plasma exposure at 450 °C and (b) after an annealing at 350–400 °C for 60 min, clearly show the flattening of the surface as a result of soft annealing (*V*_tip_ = 50 mV and *I* = 70 pA).

**Figure 5:**
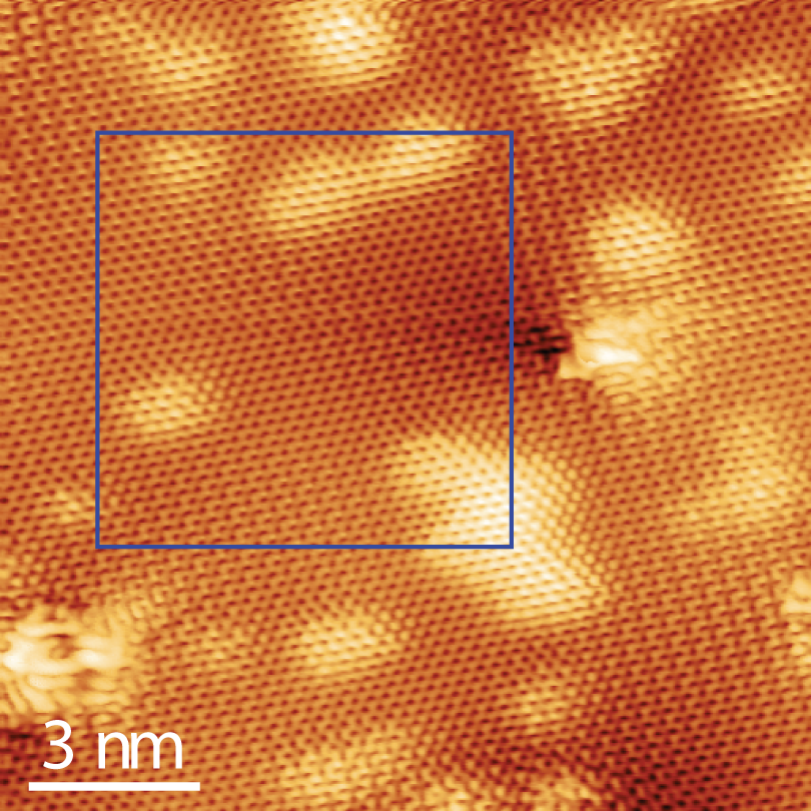
Top-view STM image of the surface consisting of various atomic-scale patterns where a very low defect density can be observed. The marked area is also shown in Figure 4b.

In order to corroborate the discussions of spectroscopy results further, scanning tunneling microscopy (STM) was utilized. STM image of pristine HOPG consists of a hexagonal pattern generated by the charge density of the electrons [37]. After exposure to hydrogen plasma, the surface still consists of a hexagonal pattern but on a highly corrugated plane (Figure 4a). Soft annealing leads to a flatter surface; however, it still has a corrugation in the form of ripples and valleys at certain points (Figure 4b). This surface corrugation matches well with the theoretical calculation of a suspended graphane layer, where it is estimated that this layer should be corrugated in the form of ripples with an amplitude of a few hundred picometers [27]. The hexagonal ring patterns in Figure 5 appear in different distorted forms. Our STM images are similar to those obtained locally around the step edges of graphite, where the step edges were terminated with atomic hydrogen [38]. Since STM probes the local density of states [37], this distortion in the ring patterns may arise from surface corrugation [38]. On the other hand, it should not be disregarded that graphane has different possible stable C–H conformations and these conformers do have distorted ring structures [3–4], which may also explain the variety of different distorted STM contrasts observed even on relatively flat surfaces. Moreover, it can be seen that same atomic patterns can be observed on both light and dark contrast sites.

The STM results confirm that we have obtained a new network of carbon atoms on a rippled plane, different from surface [20] and local step-edge hydrogenations [38]. However, it should be mentioned that before the introduction of hypothetical graphane in 2007, a very similar hydrogen LTP exposure to ours was performed by Ruffieux et al. [39]. With the STM method, they recorded very similar current patterns. They attributed these patterns solely to defects induced by the hydrogen plasma. With respect to this point, STM method by itself is not conclusive enough to substantiate hydrogenation of HOPG.

## Conclusion

(1) Hydrogen-LTP-exposed HOPG and graphene are characterized with various techniques including photoelectron spectroscopy, Raman spectroscopy and scanning probe microscopy. The hydrogen-LTP-exposed HOPG surface consists of various atomic-scale STM patterns, which may be due to different possible C–H conformations of hydrogenated graphene layers. On the other hand, surface corrugation or point defects caused after LTP exposure also have a contribution to these patterns. Regarding its valence-band structure measured with UPS, hydrogen-LTP-exposed HOPG has similar features to cubic diamond. Raman spectroscopy of hydrogen-LTP-treated single-layer graphene reveals a D peak to G peak ratio of more than 4, which is due to hydrogenation. Graphane is a reserved word for graphene that is hydrogenated from both sides. Though such a case cannot be perfectly realized due to the hydrogen deficiencies and point defects created during the plasma treatment, our results, when considered all together, point to double-sided hydrogenation of the graphene layers.

(2) Graphite may be an alternative solution for hydrogen storage. Since hydrogen-LTP-exposed HOPG possesses a high thermal stability, unloading of chemically stored hydrogen requires annealing over 1000 °C which may not be very feasible. However, hydrogen gas which had caused blisters on the surface may still be stuck between the graphane layers, where the required unloading temperature is around 450 °C.

## Experimental

### Plasma creation and exposure

The experiments were conducted in the plasma exposure facility at the University of Basel [40]. Plasma was created 75 cm away from the sample in a Pyrex tube through a matching network by a 13.56 MHz RF generator at a typical power of 49 W. This RF power was coupled to the tube by an outer electrode acting as a surfatron [41]. The plasma source was mounted onto a metallic UHV chamber, where a metallic carousel with heatable and biasable sample holders is situated at the heart of this chamber. Hydrogen (6.0 purity) at a pressure of 3 Pa was used, where the background pressure was 5 × 10^−6^ Pa. Optical emission spectrum of the plasma yields no peaks other than those of hydrogen and self-ionized mass spectroscopy does not reveal any other ions than those of hydrogen (not shown here). The hydrogen plasma was characterized with a commercial Hiden ESPion Langmuir probe. The probe measurements were performed 5 cm away from the sample where an average *T*_e_ of 3.5 ± 0.5 eV and an ion flux of 1.5 ± 0.5 × 10^15^ cm^−2^s^−1^ were obtained. The exact plasma chemistry of the hydrogen ions (H^+^, H_2_^+^, H_3_^+^) was not known, therefore an estimate of 2 a.m.u. was used as the average ion mass, which introduces ≈10% uncertainty to the *T*_e_ calculation. ε*_i_* was calculated as a sum of the energy gained on traversing the plasma sheath (from balancing ion and electron fluxes at the sample surface) and the initial ion energy: ε_i_ = (*T*_e_/2)ln(*M*/2π*m*) + 0.5(*T*_e_) which corresponds to 3.34 *T*_e_ for H^+^ and 3.88 *T*_e_ for H_3_^+^ ions. For simplicity, ε_i_ was taken as 3.6 *T*_e_, which is 12.6 ± 1.8 eV in our case. We think that double-sided hydrogenation may be achieved under these plasma conditions. It is also possible to achieve single-surface hydrogenation, anisotropic chemical etching [28] or physical sputtering of HOPG by changing the plasma parameters or sample conditions.

Implantation is a homogeneous process taking place all over the HOPG subsurface layers. With a pessimistic approach, one can assume that only 10% of the impinging ions are atomic, where half of them become backscattered, physisorbed or chemisorbed on the surface, leaving only 7.5 × 10^13^ cm^−2^s^−1^ of them becoming implanted as atomic hydrogen. Also taking the H–H combinations in the HOPG into account, the necessary ion fluence would correspond to several minutes to hydrogenate the subsurface layers of the HOPG, where the surface density is 3.8 × 10^15^ cm^−2^ and hydrogen uptake is 1:1 for the graphane formation.

During hydrogen plasma exposure, samples were electrically floating while they were being heated resistively. HOPG samples were cleaved in air before they were introduced to the UHV environment. Temperature calibration was done in a separate exposure by using a chromel alumel thermocouple mounted on the sample and being controlled with a pyrometer during actual exposures.

### Characterization methods

Raman spectroscopy measurements were performed by using a WITec alpha 300 confocal Raman microscope. The wavelength of the excitation laser was 532 nm and the power of the laser was kept at 2.1 mW without noticeable sample heating or damaging. The laser spot size was 360 nm at 100× magnification. The spectral resolution was 3 cm^−1^ and each spectrum was an average of 20 accumulations with an integration time of 0.5 seconds per accumulation. Graphene flakes for Raman spectroscopy measurements were prepared by exfoliation of HOPG using a PDMS stamp and transferring them on SiO_2_ [42]. The number of layers was determined from the 2D peak of the Raman spectra, which was acquired prior to plasma treatment.

An intermittent contact-mode AFM experiment was performed by means of a Nanosurf FlexAFM operated in ambient conditions. The quantities that were measured are the cantilever oscillation amplitude (*A*_free_ = 20 nm) and phase related to the driving signal. The distance to the sample was controlled in a feedback loop, maintaining the cantilever oscillation amplitude equal to a given setpoint value (typically 0.5–0.65 · *A*_free_). The topography image was acquired by changing the *xy* position of the cantilever tip over the scanned surface. The full range of the scanner was equal to 10 μm × 10 μm. A commercially available Nanosensors PPP-NCLPt silicon cantilever was used. The fundamental frequency, spring constant, and quality factor of the cantilever were equal to *f*_0_ = 142 kHz, *k* = 20 N/m, *Q* = 300, respectively. We avoided performing electron microscopy on the HOPG samples because the electron beam energy could ionize H_2_O and NH_3_ adsorbents and cause additional effects [43].

XPS measurements were performed under UHV conditions with a VG ESCALAB 210 spectrometer by using monochromated Al Kα radiation (1486.6 eV) with an energy resolution better than 0.5 eV. A helium discharge lamp emitting in the ultraviolet range (He I, 21.2 eV) was used for UPS measurements. The samples were transferred to the photoemission chamber without breaking the high-vacuum conditions. The base pressure in the chamber was around 1 × 10^−7^ Pa during acquisition. A normal electron escape angle (i.e., polar angle = 0°) and a step size of 0.025 eV were used. The Au 4f_7/2_ line was measured at a binding energy of 83.78 eV, hence all our XPS peaks are shifted by −0.22 eV. Wide-scan XPS spectra from 0 to 1200 eV showed only carbon, which precludes a possible interpretation of the results as a reaction with an unknown element.

STM was performed with a commercial qPlus STM/AFM microscope (Omicron Nanotechnology GmbH) at 77 K under UHV conditions and operated by a Nanonis Control System from SPECS GmbH. All STM images were recorded at constant current mode with the bias voltage applied to the tip. The samples were heated to 80–400 °C prior to measurements in UHV.
